# Changes in Atopic Sensitisation From 1 to 5 Years of Age: Longitudinal Data From the MIS BAIR Trial

**DOI:** 10.1111/cea.70144

**Published:** 2025-09-22

**Authors:** Nicole L. Messina, Laure F. Pittet, Emily K. Forbes, Kaya Gardiner, Katie L. Flanagan, Anne‐Louise Ponsonby, Roy Robins‐Browne, Frank Shann, Mike South, Peter Vuillermin, Susan Donath, Dan Casalaz, Nigel Curtis, the Melbourne Infant Study: BCG for Allergy and Infection Reduction (MIS BAIR) Group

**Affiliations:** ^1^ Infectious Diseases Group Murdoch Children's Research Institute Parkville Victoria Australia; ^2^ Department of Paediatrics The University of Melbourne Parkville Victoria Australia; ^3^ Immunology, Vaccinology, Rheumatology and Infectious Diseases Unit Geneva University Hospitals and Faculty of Medicine Geneva Switzerland; ^4^ Department of Research Operations The Royal Children's Hospital Melbourne Parkville Victoria Australia; ^5^ Westmead Hospital Sydney New South Wales Australia; ^6^ School of Medicine University of Tasmania Hobart Tasmania Australia; ^7^ School of Health and Biomedical Science RMIT University Melbourne Victoria Australia; ^8^ Population Allergy Murdoch Children's Research Institute Parkville Victoria Australia; ^9^ The Florey Institute for Neuroscience and Mental Health University of Melbourne Parkville Victoria Australia; ^10^ Department of Microbiology and Immunology, Peter Doherty Institute for Infection and Immunity The University of Melbourne Parkville Victoria Australia; ^11^ Department of General Medicine The Royal Children's Hospital Melbourne Parkville Victoria Australia; ^12^ Institute of Mental and Physical Health and Clinical Translation Deakin University Geelong Victoria Australia; ^13^ Child Health Research Unit Barwon Health Geelong Victoria Australia; ^14^ Clinical Epidemiology & Biostatistics Unit Murdoch Children's Research Institute Parkville Victoria Australia; ^15^ Neonatal Intensive Care Unit Mercy Hospital for Women Heidelberg Victoria Australia; ^16^ Department of Infectious Diseases The Royal Children's Hospital Melbourne Parkville Victoria Australia

**Summary**

- At 5 years of age, 37% of children were sensitised to at least one allergen.
- From 1 to 5 years of age, atopic sensitisation changed from predominantly food‐related to aeroallergen‐related.
- Persistence of food sensitisation ranged from 6.3% to 37%, depending on the specific allergen.

To the Editor,

Vaccination with BacilleCalmette‐Guérin (BCG) has off‐target effects on the immune system which may influence the risk of developing allergic disease in later life. The Melbourne Infant Study: BCG for Allergy and Infection Reduction (MIS BAIR) randomised controlled trial (NCT01906853) investigated whether neonatal BCG vaccination reduces the incidence of allergy, eczema and infections at 1 and 5 years of age, and asthma at 5 years of age [1]. In the trial, neonatal BCG vaccination reduced the incidence of eczema at 1 year [2], particularly in children of parents with a history of eczema, but did not significantly reduce lower respiratory tract infections [3] or food allergy [4] at 1 year of age. At 5 years of age, neonatal BCG vaccination reduced the risk of more severe forms of asthma but had minimal effect on the overall incidence of asthma [5].

The MIS BAIR trial also aimed to investigate atopic sensitisation to a range of common allergens at 5 years of age. This required participants to attend a clinic visit for skin prick testing (SPT) at 5 years of age [1, 4]. In March 2020, COVID‐19 pandemic ‘lockdown’ measures in Melbourne (which included stay‐at‐home orders [6, 7]) prevented 5‐year SPT clinic visits being done for the following 12 months. During this period, 1124 (88%) of participants were eligible for their SPT clinic visit, with 62.7% (798/1272) only eligible after March 2020. To provide an opportunity for these participants to attend their SPT clinic visit after lockdown restrictions had eased, the age of eligibility for the SPT clinic visit was extended to 7 years of age. However, participants' families remained reluctant to visit a hospital setting during the pandemic and consequently, overall, only 34.2% (435/1272) attended a school‐age (5–7 years of age) SPT clinic visit (Figure 1A).

**FIGURE 1:**
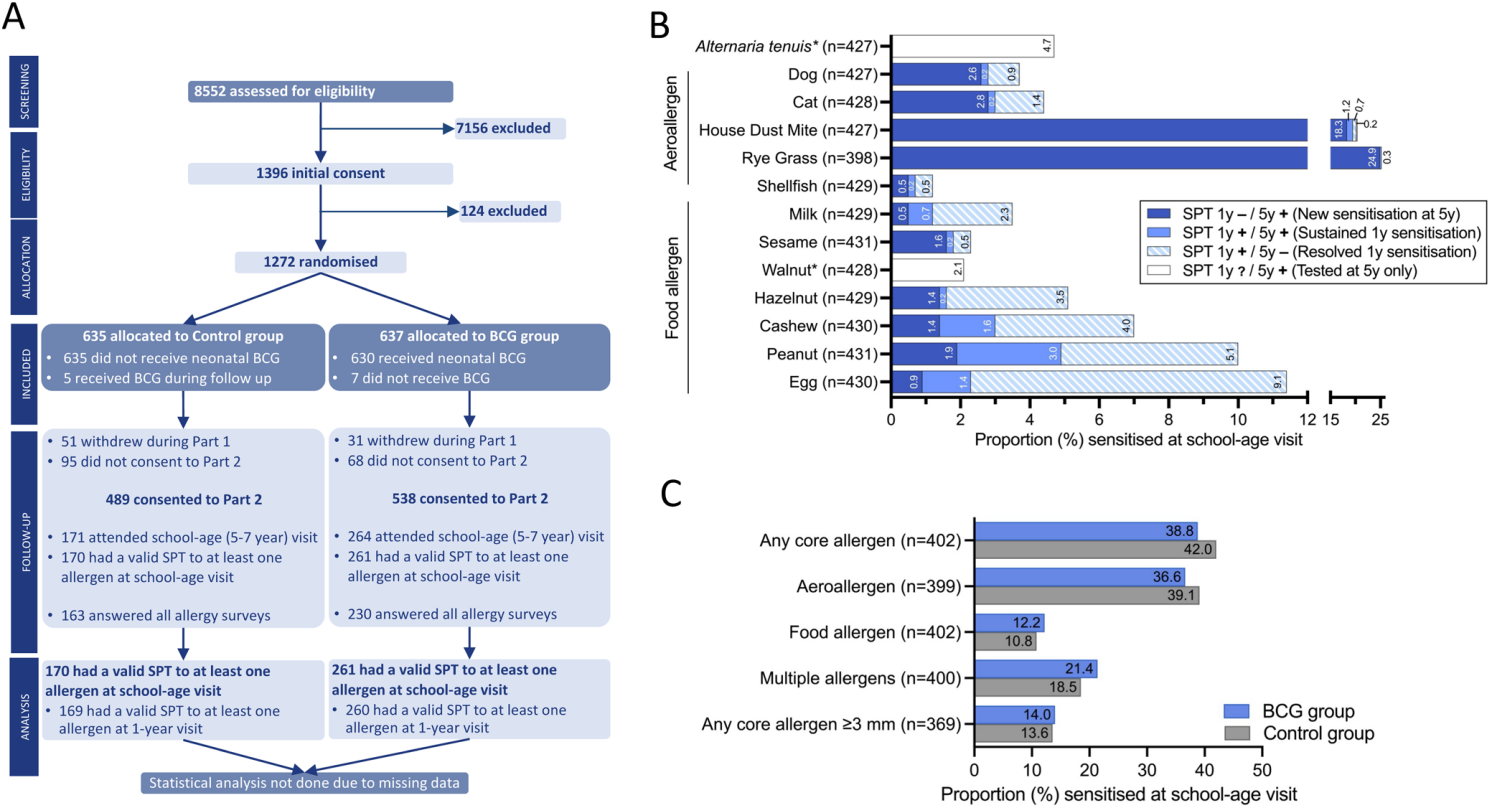
Atopic sensitisation at 5–7 years. (A) CONSORT diagram for MIS BAIR atopic sensitisation at school‐age visit (5–7 years). Written/electronic consent was obtained from a parent/guardian prior to randomisation and for inclusion in Part 2. (B) Atopic sensitisation (allergen wheal ≥ 2 mm above negative control) for each core allergen at the 1‐year and school‐age (5–7‐year) visits for children who had a SPT at the school‐age visit. SPT 1 y ?/5 y + indicates allergens that were not tested or for which SPT was indeterminant at the 1‐year visit and were SPT positive at the school‐age visit. * indicates allergens only included as core allergens at the school‐age visit. (C) Atopic sensitisation at the school‐age visit by randomisation group. Data presented as a proportion of participants with non‐missing data for each outcome (i.e., positive for at least one allergen or non‐missing for all core allergens). BCG, Bacille Calmette‐Guérin; SPT, skin prick test; y, year.

In addition to the low follow‐up rate, there was a difference between the randomisation groups in the proportion of participants who did not attend an SPT clinic visit (58.6% (373/637) in the BCG group vs. 73.1% (464/635) in the Control group) (Figure 1A). This might be attributable to disappointment in being randomised to the Control group [8] as participants' families were not blinded to the randomisation group due to BCG vaccination site scarring. Despite the large proportion of missing data, the available SPT results and longitudinal testing provide valuable information in relation to the progression of atopic sensitisation in early life.

Among the 435 participants who attended, the median age at the SPT clinic visit was 64 months (IQR 61–68 months; range 60–87 months). Of these, 99.1% (431/435) had a valid SPT, and 90.6% (394/435) were tested against all the pre‐specified food (peanut, cashew nuts, hazelnut, walnut, raw egg, cow's milk, sesame and shellfish) and aero (dog, cat, house dust mite, rye grass and *Alternaria tenuis* ) allergens. Among participants with a valid SPT, 37.4% (161/431) had atopic sensitisation (wheal ≥ 2 mm above negative control) to at least one allergen. The majority of these participants had sensitisation to rye grass (62.1%, 100/161) and/or house dust mite (52.8%, 85/161) (Figure 1B). Sensitisation to more than one allergen was found in 19.0% (81/426) of participants with a valid SPT across multiple allergens, with 48 participants sensitised to 2, 16 to 3, 10 to 4 and 7 to 5 or more allergens, mostly grass (7/7), house dust mite (6/7), peanut (5/7), and egg (5/7).

Consistent with previous studies [9], sensitisation to food allergens decreased over time from 18.2% (198/1089) at 1 year to 10.9% (47/431) at 5–7 years of age (school‐age). This resulted from both fewer new and resolution of prior food sensitisation (Figure 1B). Persistence of sensitisation to food allergens between the one‐year and school‐age SPT visits varied between foods. Egg sensitisation decreased from 9.9% at 1 year to 2.3% at 5–7 years with only 13.3% of participants having persisting sensitisation. Similarly, hazelnut sensitisation only persisted for 6.3% of participants. For other food allergens, sensitisation persisted for 23%–37% of participants between the one‐year and school‐age visits (milk 23.1%, cashew 29.2%, sesame 33.3%, shellfish 33.3% and peanut 37.1%). In contrast, sensitisation to aeroallergens (rye grass, house dust mite, dog and cat) increased from an incidence of 5.0% (54/1089) at 1 year [4] to 34.8% (150/431) at 5–7 years of age (Figure 1B).

The incidence of atopic sensitisation to any core allergen was 38.8% in the BCG group (95/245) and 42.0% in the Control group (66/157) (Figure 1C). Given the large proportion of missing data and the imbalance between intervention groups, statistical comparison of the effects of BCG vaccination on atopic sensitisation at 5–7 years of age was not feasible.

We investigated the factors associated with non‐attendance at the school‐age visit and, as expected, found that it was associated with the visit being due after 1 March 2020 (*χ* ^2^ *p* < 0.001) and being randomised to the Control group (*χ* ^2^ *p* < 0.001). It was also associated with lower maternal education (*χ* ^2^ *p* < 0.001), attending childcare in the first year of life (*χ* ^2^ *p* = 0.005) and living more than 30 km from the clinic (*χ* ^2^ *p* = 0.02). Non‐attendance at the school‐age visit was not associated with other variables tested, including atopic sensitisation or eczema at 1 year of age, family history of allergic disease (allergy, eczema, hay fever or asthma) or parents born overseas (data not shown).

These data provide valuable insights into the evolution of sensitisation to both food and aeroallergens during early childhood in Melbourne, a setting with a high prevalence of allergic disease.

## Author Contributions

N.C. was the lead investigator and responsible for study conception, design and funding acquisition. N.C. and S.D. developed the final scientific protocol and ethics application, and all other authors provided critical evaluation and revision. K.G. co‐ordinated, and N.C., D.C., P.V. and N.L.M. were involved in implementation. E.K.F. and N.L.M. developed, and N.C., S.D., L.F.P. and M.S. contributed to the analysis plan. E.K.F. led, N.L.M. supervised and L.F.P. contributed to data cleaning and preparation. N.L.M. drafted the manuscript and co‐ordinated manuscript preparation and revision. All authors provided critical evaluation and revision of the manuscript.

## Conflicts of Interest

The authors declare no conflicts of interest.

## Data Availability

The data that support the findings of this study are available from the corresponding author upon reasonable request.
